# Propranolol Sensitizes Vascular Sarcoma Cells to Doxorubicin by Altering Lysosomal Drug Sequestration and Drug Efflux

**DOI:** 10.3389/fonc.2020.614288

**Published:** 2021-02-01

**Authors:** Jhuma Saha, Jong Hyuk Kim, Clarissa N. Amaya, Caleb Witcher, Ali Khammanivong, Derek M. Korpela, David R. Brown, Josephine Taylor, Brad A. Bryan, Erin B. Dickerson

**Affiliations:** ^1^Department of Veterinary Clinical Sciences, College of Veterinary Medicine, University of Minnesota, St. Paul, MN, United States; ^2^Animal Cancer Care and Research Program, College of Veterinary Medicine University of Minnesota, St. Paul, MN, United States; ^3^Masonic Cancer Center, University of Minnesota, Minneapolis, MN, United States; ^4^Department of Biomedical Sciences, Texas Tech University Health Sciences Center, El Paso, TX, United States; ^5^Paul L. Foster School of Medicine, Texas Tech University Health Sciences Center, El Paso, TX, United States; ^6^Department of Biology, Stephen F. Austin State University, Nacogdoches, TX, United States; ^7^Department of Veterinary and Biomedical Sciences, College of Veterinary Medicine, University of Minnesota, St. Paul, MN, United States

**Keywords:** angiosarcoma, hemangiosarcoma, propranolol, doxorubicin, drug resistance, lysosome

## Abstract

Angiosarcoma is a rare cancer of blood vessel–forming cells with a high patient mortality and few treatment options. Although chemotherapy often produces initial clinical responses, outcomes remain poor, largely due to the development of drug resistance. We previously identified a subset of doxorubicin-resistant cells in human angiosarcoma and canine hemangiosarcoma cell lines that exhibit high lysosomal accumulation of doxorubicin. Hydrophobic, weak base chemotherapeutics, like doxorubicin, are known to sequester within lysosomes, promoting resistance by limiting drug accessibility to cellular targets. Drug synergy between the beta adrenergic receptor (β-AR) antagonist, propranolol, and multiple chemotherapeutics has been documented *in vitro*, and clinical data have corroborated the increased therapeutic potential of propranolol with chemotherapy in angiosarcoma patients. Because propranolol is also a weak base and accumulates in lysosomes, we sought to determine whether propranolol enhanced doxorubicin cytotoxicity *via* antagonism of β-ARs or by preventing the lysosomal accumulation of doxorubicin. β-AR-like immunoreactivities were confirmed in primary tumor tissues and cell lines; receptor function was verified by monitoring downstream signaling pathways of β-ARs in response to receptor agonists and antagonists. Mechanistically, propranolol increased cytoplasmic doxorubicin concentrations in sarcoma cells by decreasing the lysosomal accumulation and cellular efflux of this chemotherapeutic agent. Equivalent concentrations of the receptor-active S-(−) and -inactive R-(+) enantiomers of propranolol produced similar effects, supporting a β-AR-independent mechanism. Long-term exposure of hemangiosarcoma cells to propranolol expanded both lysosomal size and number, yet cells remained sensitive to doxorubicin in the presence of propranolol. In contrast, removal of propranolol increased cellular resistance to doxorubicin, underscoring lysosomal doxorubicin sequestration as a key mechanism of resistance. Our results support the repurposing of the R-(+) enantiomer of propranolol with weak base chemotherapeutics to increase cytotoxicity and reduce the development of drug-resistant cell populations without the cardiovascular and other side effects associated with antagonism of β-ARs.

## Introduction

Angiosarcoma is an extremely rare (0.01% of all cancers) and highly aggressive malignancy of blood vessel forming cells with few effective treatment options and a high incidence of patient mortality (1–5). The median overall survival time for local disease is approximately 30–50 months, but for patients with metastatic disease, survival drops to approximately 10–12 months (6–8). Treatment for local disease involves wide surgical resection, with the option of radiotherapy, and the addition of adjuvant chemotherapy for patients with nonresectable tumors or metastatic disease (2). Despite these aggressive approaches and initial responses produced by anthracycline-based regimens or taxanes, most angiosarcomas eventually become resistant to chemotherapy (9, 10). Identification of the mechanisms used by angiosarcomas to evade chemotherapies, and the pursuit of approaches to override these mechanisms remains a key strategy to improve patient survival.

Due to the rare occurrence of angiosarcoma, undertaking extensive studies to identify and dissect treatment resistance mechanisms that develop in these tumors remains a challenge. Hemangiosarcoma is a common cancer in dogs that closely models the genetic landscape and pathogenesis of human angiosarcoma and follows a similar clinical course (11). Survival times remain short for most dogs, with a median survival of 4 to 6 months when treated with the standard of care of surgery followed by adjuvant chemotherapy (12–14). Although initial responses to chemotherapy are often observed, the development of drug resistance along with disease progression eventually follow. Hence, canine hemangiosarcoma provides a relevant clinical model to study and identify drug resistance mechanisms for human angiosarcoma.

We previously identified a subset of cells in human angiosarcoma and canine hemangiosarcoma cell lines that is highly resistant to doxorubicin due to its accumulation within lysosomes (15). Weakly basic, lipid-soluble compounds can accumulate in lysosomes through a process known as lysosomal sequestration or trapping and are referred to as lysosomotropic molecules (16, 17). Chemotherapies are often formulated as weakly basic amines, and many of these drugs have been shown to undergo lysosomal sequestration (18–21). Lysosomal accumulation of chemotherapies occurs by their passive diffusion across cell membranes, where they are rapidly protonated due to the acidic environment of the lysosomes (16, 17). Protonated molecules are less able to move back across the membrane and out of the lysosome due to their decreased membrane permeability and remain trapped inside the acidic lumen. As a result, the sequestered drugs fail to reach their target sites and exert a cytotoxic effect (22–24).

Drug resistance due to lysosomal sequestration is both an intrinsic cellular feature as well as an acquired response to drug treatment. Naïve cancer cell lines harboring a higher number of lysosomes were more inherently resistant to the cytotoxic effects of drug treatment compared to those with lower numbers (25). In contrast, development of resistance occurred after exposure of cancer cell lines to several cytotoxic drugs known to sequester within lysosomes (25). These drugs induced lysosome biogenesis, which is regulated through the activation of the transcription factor EB (TFEB) (25). Drug-induced activation of TFEB initiated the transcription of genes responsible for lysosome biogenesis, allowing cells to increase lysosome numbers as well as lysosomal volume, further enhancing lysosomal drug sequestrations and drug resistance (25). Developing strategies to reduce lysosomal drug sequestration of chemotherapy drugs in angiosarcoma and hemangiosarcoma represents a viable approach to overcome this mode of chemoresistance in these tumors.

The β-AR antagonist, propranolol, contains a weakly basic amine moiety and has been shown to accumulate in lysosomes (26). The drug exists as a racemic mixture, consisting of equal amounts of its receptor-active S-(−) and -inactive R-(+) enantiomers (27). Originally developed as an anti-hypertensive drug and currently indicated for the treatment of hypertension, arrhythmia, ischemic heart disease, anxiety, and migraines, a number of preclinical studies and clinical reports suggest that propranolol can be repurposed for the treatment of angiosarcoma (28–34). This application stemmed from the serendipitous discovery (35) and positive findings from a subsequent clinical trial (36) that propranolol could be used to treat infantile hemangioma, a benign vascular tumor. This discovery has led to the repurposing of propranolol as the gold standard for the management of problematic hemangiomas (36, 37). Although propranolol has been used as a single agent to treat infantile hemangioma, it has been combined with various chemotherapies and found to synergize with doxorubicin, paclitaxel, and vinblastine to enhance the antiproliferative and antiangiogenic properties of these drugs in cancer cell lines and to increase their anti-tumor efficacy in mouse models of triple negative breast cancer and neuroblastoma (32, 38, 39). Several studies have evaluated the efficacy of propranolol in combination with chemotherapy in angiosarcoma patients (28–32, 34). In one study, treatment with propranolol and the chemotherapeutic *Vinca* alkaloid, vinblastine, resulted in partial or complete responses in a group of seven patients with metastatic or recurrent angiosarcoma and reported an increase in the median overall survival of 16 months (32). Partial and complete responses have also been described in a handful of patients presented as individual case studies, further bolstering support for the use of propranolol in combination with chemotherapy for the treatment of localized and advanced disease (28–31).

Here, we report that propranolol synergizes with doxorubicin *in vitro*, independent of its β-AR antagonism but consistent with its properties as a weakly basic lysosomotropic drug. Our results suggest that by targeting the lysosomal compartment, propranolol reduces the sequestration of doxorubicin within lysosomes while simultaneously blocking doxorubicin efflux and increasing its total intracellular accumulation. Understanding how propranolol promotes synergy with doxorubicin or other chemotherapies could be used to develop new strategies to overcome chemoresistance and to improve treatment outcomes for human angiosarcoma and canine hemangiosarcoma.

## Materials and Methods

### Cell Culture

The COSB canine hemangiosarcoma cell line was derived from a xenograft of the original cell line, SB-HSA (40), and the DD-1 cell line was derived from a splenic hemangiosarcoma (41). The human angiosarcoma cell line, ISO-HAS (42) was kindly provided by Dr. Mamiko Masuzawa (Kitasato University School of Medicine, Japan), and the mouse angiosarcoma cell line, SVR (43, 44), was purchased from the American Type Culture Collection (CRL-2280, Manassas, VA, USA). All cell lines were grown and maintained in endothelial cell growth medium (41, 45, 46) for approximately 8–10 weeks before new vials were thawed to ensure similar passage numbers were used for the experiments. All cell lines were tested for mycoplasma and authenticated by IDEXX Laboratories (Westbrook, ME, USA) to verify cell line purity and quality.

### Immunohistochemistry

Immunohistochemistry (IHC) was performed on 4 µm sections of formalin-fixed, paraffin-embedded samples as described previously with some modifications (33, 47). Sections were dewaxed in xylene and hydrated in graded ethanol. Endogenous peroxidase activity was blocked with 0.3% H_2_O_2_ in distilled water for 20 min at room temperature, followed by three washes in phosphate buffered saline (PBS; pH 7.4, 137 mM NaCl, 2.7 mM KCl, 10 mM Na_2_HPO_4_, 2 mM KH_2_PO_4_) with 0.1% Tween^®^ 20 (PBST). Antigen retrieval was attained by boiling tissue sections in citric acid buffer (pH 6.0) for 10 min in a microwave oven (700 W, high power). After cooling, the slides were washed three times in PBST. Sections were then incubated with primary antibodies at room temperature for 2 h. The primary antibodies used against epitopes in the β-ARs were rabbit anti-β1-AR (1:200, #250919, Abbiotec, San Diego, CA, USA; recognizing a sequence within the center region of human β1-AR), rabbit anti-β2-AR (1:200, #251604, Abbiotec; recognizing a sequence within the center region of human β2-AR), and rabbit anti-β3-AR (1:200, #251434, Abbiotec; recognizing a sequence within the C-term region of human β3-AR). The antibodies for β1-AR and β2-AR were reported on the vendor website to recognize the analogous canine receptor proteins, and reactivity was confirmed by immunoblotting using human and canine cell lines. Primary antibody binding was detected by the use of EXPOSE Mouse and Rabbit Specific HRP/DAB Detection IHC Kit (#ab80436, Abcam, Cambridge, UK; for anti-β1-AR, anti-β2-AR, and anti-β3-AR). Sections were counterstained with Harris hematoxylin (#3530-16, RICCA Chemical Company, Arlington, TX, USA). Rabbit IgG antibody (#31235, Thermo Fischer Scientific, Waltham, MA, USA) was used as a negative isotype control. Immunostaining was assessed semi-quantitatively at high power magnification (400X). The percentage of immunoreactive cells was scored 0 to 3+, where 0 reflects specific staining in < 1% of the cells, 1+ reflects specific staining in < 25% of the cells, 2+ reflects specific staining in 25–75% of the cells, and 3+ reflects specific staining in > 75% of the cells. The intensity was assessed as weak, moderate, or strong. Immunostaining results were scored by multiplying the percentage of positive cells by the intensity.

### Immunoblotting

Immunoblotting was performed based on standard techniques (48, 49). Briefly, protein samples were collected as indicated for each experiment, subjected to SDS-polyacrylamide gel electrophoresis, and transferred to a nitrocellulose membrane or polyvinylidene fluoride (PVDF) membrane. Membranes were blocked with 50% Odyssey^®^ Blocking Buffer (#927-50000, LI-COR Biosciences, Lincoln, NE, USA) diluted in TBST (20 mM Tris– HCl, pH 7.4, 137 mM NaCl, 0.1% Tween 20) or TBST plus 3% bovine serum albumin (BSA). After blocking, the membranes were incubated overnight at 4°C with one of the following antibodies diluted in Odyssey^®^ Blocking Buffer or TBST +3% BSA: anti-β1-AR (1:1000, #250919, Abbiotec), anti-β2-AR (1:1000, #251604, Abbiotec), anti-β3-AR (1:1000, #251434, Abbiotec), anti-phospho-checkpoint kinase 1 (CHK1) (Ser345) (1:1000, #2348, Cell Signaling, Danvers, MA, USA), anti-phospho-checkpoint kinase 2 (CHK2) (Thr68) (1:1000, #2197, Cell Signaling), anti-phospho-ataxia telangiectasia- and Rad3-related protein (ATM) (Ser428) (1:1000, #2853, Cell Signaling), anti-phospho-protein kinase ataxia-telangiectasia mutated protein (ATR) (Ser1981) (1:1000, #5883, Cell Signaling), and anti-β-actin (1:5000, #sc8432, Santa Cruz Biotechnology, Dallas, TX, USA; 1:5000, #A5441, Sigma-Aldrich, St. Louis, MO, USA). After incubation with primary antibodies, the membranes were washed three times with TBST, followed by incubation with LI-COR IRDye 800CW (780 nm) donkey anti-rabbit (1:10,000-1:20,000, #925-32213, LI-COR Biosciences) and IRDye 680RD (680 nm) donkey anti-mouse (1:10,000–1:20,000, #925-68072, LI-COR Biosciences) infrared fluorescence dye conjugated secondary antibodies for 60 min at room temperature. The membranes were washed 3 times with TBST followed by one wash with TBS (without Tween 20). The membranes were then scanned and documented using an Odyssey infrared imaging system (LI-COR Biosciences) at 680 nm and 780 nm emission wavelengths. For membranes blocked with TBST and 3% BSA, primary antibodies were detected using HRP-conjugated secondary antibody, subjected to Supersignal West Dura Extended Duration Substrate (#34075, Thermo Fisher Scientific, Waltham, MA, USA) and digitally captured using a GE Image Quant Las4000 imaging system.

### Phospho-Antibody Array

COSB cells were treated with epinephrine (1 µM) (#E4642, Sigma Aldrich), propranolol (100 nM) (#0624, Tocris, Minneapolis, MN, USA), or phentolamine (100 nM) (#6431, Tocris) alone or in combination for 30 min, and lysates were analyzed using the Phospho-Mitogen-Activated Protein Kinase (MAPK) Antibody Array (#ARY002B, R&D Systems, Minneapolis, MN, USA) as described (34, 49). Densitometry of each antibody array signal was performed using ImageJ software. Reference spots on each array were used to normalize the pixel densities. The numerical protein expression data was normalized, and centroid linkage based on an uncentered correlation similarity metric was performed using Gene Cluster 3.0 software. Heatmaps were generated using Java TreeView software.

### Cell Viability Assay

Cells were plated in triplicate at 7,000-10,000 cells per well in 96-well plates in 100 µL of cell culture medium and exposed to increasing concentrations of doxorubicin (Bedford Laboratories, Bedford, OH, USA), propranolol, or the S-(−) (#0834, Tocris) or R-(+) (#0835, Tocris) enantiomers of propranolol, as indicated. For assays using LysoTracker^®^, cells were plated as above with 50 nM LysoTracker Deep Red (#L12492, Thermo Fisher Scientific). Cell viability was determined 72 h later using a colorimetric MTS reagent (Cell Titer 96^®^ Aqueous Non-Radioactive Cell Proliferation Assay, Promega, Madison, WI, USA), according to the manufacturer’s instructions. The relative median lethal concentration (LC50) values were determined using GraphPad Prism 6 software (GraphPad Software Inc., La Jolla, CA, USA). For the drug synergy studies, cells were plated as above and allowed to adhere overnight. Cells were treated with combinations of doxorubicin (1–250 nM) and propranolol (3–100 µM) for 72 h and viability was determined as described above. Combination index (CI) values for all tested drug concentrations were determined according to the method of Chou and Talalay (50) and calculated using Compusyn software (Combosyn Inc., NJ, USA), as described (51, 52). The CI theorem quantitatively defines the CI values as additivity (0.9 ≤ CI ≤ 1.1), synergy (CI < 0.9), and antagonism (CI > 1.1).

### LysoTracker Deep Red Assay

Cells were harvested by trypsinization, plated at 50,000 per well in 96 well plates, and allowed to adhere overnight. The next day, cells were washed with PBS and incubated with 50 nM LysoTracker Deep Red and increasing concentrations of propranolol in serum free medium, on ice for 30 min. The cells were washed, and the relative levels of LysoTracker Deep Red were detected using a TECAN Infinite m200 PRO plate reader (ex/em 647/668) (Tecan US, Morrisville, North Carolina).

### DCV Efflux Assay

DyeCycle Violet (DCV) (#V35003, Thermo Fisher Scientific) efflux assays was carried out as previously described (15). Briefly, each cell line was plated in 60 mm cell culture dishes in endothelial cell growth medium and allowed to adhere overnight. The cells were washed with PBS, and low serum endothelial growth medium (2% FBS) was added to each dish. The cells were then treated with propranolol for 18 h in low serum medium, washed, and propranolol added back to the cells for 1 h. The cells were harvested using trypsin, followed by incubation with or without 50 µM verapamil for 15 min at 37°C while maintaining all the treatment conditions. DCV was added to a final concentration of 10 µM, and the cells were incubated for an additional 60 min at 37°C with intermittent mixing. Cells were washed and maintained on ice until analysis. Propidium iodide (#R37108, Thermo Fisher Scientific) was added to each sample before flow cytometry to exclude dead cells from analysis. DCV emission was detected using an LSRII flow cytometer (BD Biosciences). Verapamil, a well-known drug efflux pump inhibitor, was used to define the efflux gates. The data were analyzed using FlowJo software (Tree Star Inc., Ashland, OR, USA).

### Doxorubicin Exclusion/Retention Assay

Cells were plated at 250,000 cells per well in 6-well plates in standard cell culture medium. After the cells had adhered, the medium was changed to a low serum medium (2% FBS), and the cells were incubated for 1 h under standard conditions. The cells were treated with 1 µM doxorubicin for 1 h. The cells were then washed with PBS, harvested by trypsinization, and the relative levels of intracellular doxorubicin assessed by flow cytometry using a BD Accuri instrument (BD Biosciences, Franklin Lakes, NJ, USA). To evaluate the effects of propranolol on doxorubicin retention, 100,000 cells per well were plated in 1 mL of culture medium in 12-well cell culture plates. After the cells had adhered, the medium was changed to the low serum medium, and the cells incubated for 18–20 h. The cells were then treated with 1 µM doxorubicin for 1 h, washed, and treated with 50 or 100 µM propranolol for 24 h. Cells not treated with propranolol were used as a control. Cells were washed with PBS, harvested using trypsin, and the relative levels of doxorubicin were analyzed by flow cytometry using a BD Accuri instrument. To exclude dead cells from analysis, 7-AAD (#A1310, Thermo Fisher Scientific) was added to cells for all treatment conditions for 10 min before data acquisition.

### DNA Damage Response

COSB cells were plated in standard cell culture medium in 6-well plates at 1 x 10^6^ cells per well and allowed to adhere overnight. The next day, the cells were pretreated with 50 µM or 100 µM propranolol for 3 h, followed by treatment with 2 µM doxorubicin for 1 h in medium supplemented with 10% FBS. Cell lysates were generated as described above, and the changes in the expression levels of phospho-CHK1 (Ser345), phospho-CHK2 (Thr68), phospho-ATR (Ser428), and phospho-ATM (Ser1981) were assessed by immunoblotting.

### Transmission Electron Microscopy

Cells were fixed overnight in a 1:1 mixture of 5% glutaraldehyde (#16120, Electron Microscopy Sciences, Hatfield, PA) and PBS at 4°C. Following three washes with PBS, cells were treated with a 1:1 mixture of 2% osmium tetroxide (#19170, Electron Microscopy Sciences) and PBS for 2 h at 4°C. Samples were rinsed three times in deionized water then stored overnight at 4°C in uranyl acetate (#22400, Electron Microscopy Sciences) en-bloc stain. Following two washes with water, cells were dehydrated in a graded ethanol series, then infiltrated with Spurrs resin (#14300, Electron Microscopy Sciences) using acetone as the transitional solvent. Infiltrated samples were embedded in fresh resin which was then polymerized overnight at 70°C. Approximately 90 nm sections were cut with a diamond knife, collected on mesh grids, and post-stained with uranyl acetate and lead citrate (#17800, Electron Microscopy Sciences). Cells were photographed using a Hitachi H-7000 transmission electron microscope (Tokyo, Japan) operating at 75KeV. Images were digitized at 800 dpi and analyzed using Magnification Version 2 (Orbicule, Inc., Leuven, Belgium). The percentage of the cell cytoplasm occupied by organelles of the lysosomal system (primary lysosomes, secondary lysosomes, and lipid-laden membrane whorls) was determined for 11 cells of each treatment.

### Statistical Analysis

All data are expressed as mean ± standard deviation (S.D.) or mean ± standard error (S.E.), as applicable. The means were analyzed, as appropriate, by unpaired *t* test (two-tailed) or one-way analysis of variance (ANOVA) with GraphPad Prism 6.0 software to determine statistical significance. All LC50 values were determined as the concentration of propranolol or doxorubicin that was lethal for 50% of the cell population using GraphPad Prism.

## Results

### Propranolol Reduces Hemangiosarcoma and Angiosarcoma Cell Viability *via* β-AR-Independent Pathways

**Figure 1:**
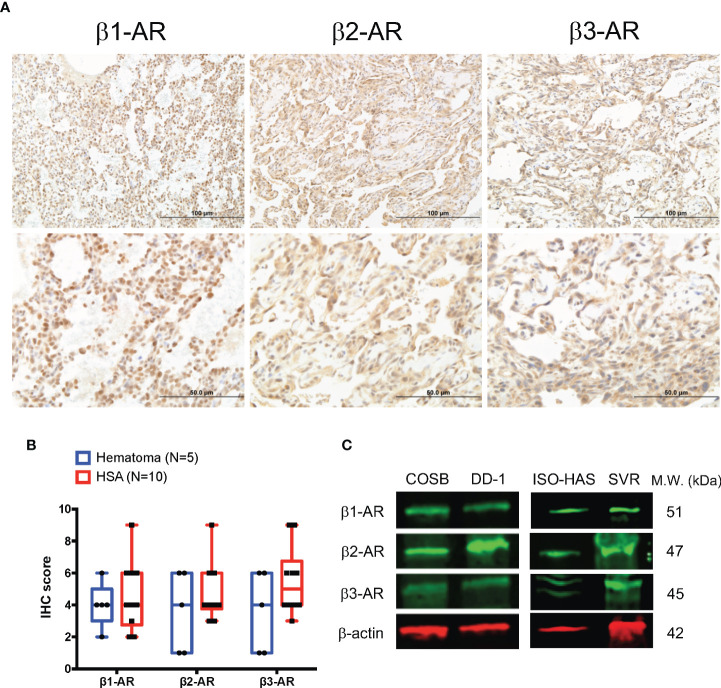
Expression of β-ARs in primary hemangiosarcomas and hemangiosarcoma cell lines. **(A)** Representative images of β1-AR, β2-AR, and β3-AR expression in visceral hemangiosarcomas (n = 10) from dogs. IHC; horseradish peroxidase; counter stain = hematoxylin. **(B)** Box and whiskers plot of β1-AR, β2-AR, and β3-AR IHC scores from the analyzed tissues and the expression in canine splenic hematomas as a control (n = 5). **(C)** Expression of β-ARs by canine hemangiosarcoma (COSB, DD-1), human angiosarcoma (ISO-HAS), and mouse angiosarcoma (SVR) cell lines. Proteins were detected in cell lysates by immunoblotting. β-actin was used as a gel-loading control.

Using IHC, we evaluated the expression of β1-, β2-, and β3-AR-like immunoreactivities in 10 samples from visceral hemangiosarcomas and sections from five, non-malignant splenic hematomas. The sample demographic characteristics are shown in **Supplemental Table S1**. Similar to our findings in human angiosarcoma (33), immunoreactivities were found against all three β-ARs in the hemangiosarcoma samples (**Figure 1A**). Immunoreactivity against β1-AR appeared to be localized to the nucleus with weak cytoplasmic staining. In contrast, immunoreactivities against β2- and β3-ARs were largely observed in the cytoplasm of these tumor cells, but some cells did show nuclear expression. Receptor-like immunoreactivities were not associated with cell membranes. Samples from splenic hematomas similarly displayed β-AR-like immunoreactivities in the cytoplasm of endothelial cells and lymphocytes (data not shown). Hemangiosarcomas appeared to express higher levels of immunoreactivity towards all three β-ARs when compared to the non-malignant hematoma samples. (**Figure 1B**; **Supplemental Table S2**). We also determined the relative levels of β-AR-like immunoreactivities in two canine hemangiosarcoma cell lines, COSB and DD-1, a human angiosarcoma cell line, ISO-HAS, and a mouse angiosarcoma line, SVR, by immunoblotting. Expression of immunoreactivities for the β-AR subtypes was detected in all four cell lines (**Figure 1C**).

To confirm that β-AR signaling is active in the cell lines, we treated COSB cells with epinephrine, which acts as both an α- and β-AR agonist, in the absence or presence of propranolol, or phentolamine, a selective α-AR antagonist. Compared to untreated controls, epinephrine treatment increased mTOR, p38 kinase, GSK-3α/β, and MKK6, but reduced the phosphorylation of Akt (**Supplemental Figure S1**). In contrast, propranolol increased the phosphorylation of Akt, a response we previously observed in angiosarcoma and breast cancer cell lines (34, 49), while phentolamine strongly increased the phosphorylation of JNK2 and CREB. Treatment of cells with epinephrine in combination with propranolol or phentolamine substantially reduced or completely eliminated the changes in phosphorylation in cells treated with epinephrine or the adrenergic receptor antagonists. These data demonstrate that α-AR and β-AR signaling occurs in COSB cells.

We and others have shown that high concentrations (> 100 µM) of propranolol alone are required to reduce hemangiosarcoma and angiosarcoma cell viability (32, 33), suggesting that the previously reported effects of propranolol on reducing tumor cell survival are not mediated by β-AR antagonism. Therefore, we treated our hemangiosarcoma and angiosarcoma cell line panel (COSB, DD-1, ISO-HAS, and SVR) with increasing concentrations of racemic propranolol, or its receptor-active S-(−) and -inactive R-(+) enantiomers (27). Racemic propranolol reduced cell viability in all of the cell lines in a concentration-dependent manner (**Table 1**; **Supplemental Figure S2**). Substantial differences in the concentrations needed to reduce cell viability were not observed between propranolol and its S-(−) and R-(+) enantiomers, or a reconstituted racemic mixture of the two enantiomers. Furthermore, assessment of viability in cell lines treated with concentrations of propranolol in the range appropriate to the affinities of the drug for β1- and β2-ARs (100 nM-1 µM) (53) did not affect cell viability (data not shown). These results suggest that the actions of propranolol on tumor cell viability are independent of β-AR expression.

**Table 1:** LC50 concentrations (µM) for propranolol and its enantiomers.

	COSB	DD-1	ISO-HAS	SVR
**Propranolol**	216.3 ± 58.6	328.4 ± 25.5	307.6 ± 27.5	115.05 ± 3.89
**R-(+)**	253.5 ± 46.6	263.8 ± 61.5	278.8 ± 12.4	138.85 ± 4.17
**S-**(−)	230.6 ± 60.1	274.3 ± 82.1	294.7 ± 8.1	125.15 ± 6.29
**R+S**	222.3 ± 41.2	349.2 ± 39.8	261.3 ± 21.1	121.2 ± 6.22

### Propranolol Increases the Sensitivity of Cells to Doxorubicin by Altering Drug Sequestration in Cellular Lysosomes

**Figure 2:**
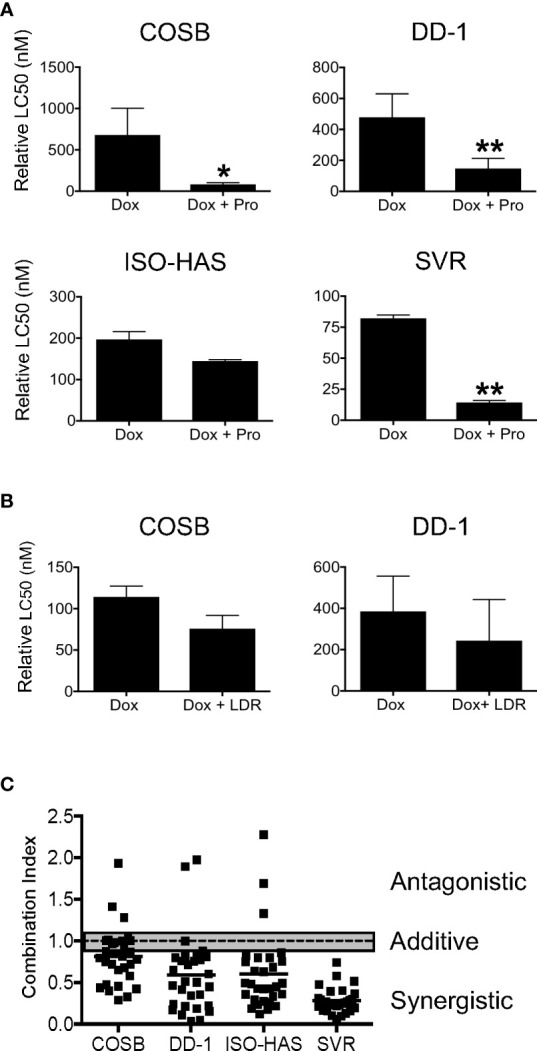
Propranolol synergizes with doxorubicin *in vitro*. **(A)** Cell viability assays were performed on COSB, DD-1, ISO-HAS, and SVR cells after 72 h of incubation with doxorubicin or doxorubicin with propranolol. Percent cell viability was determined by comparing the survival of treated wells to untreated controls. The LC50 values were determined using Prism software. Data are presented as the means ± S.D. of two to five independent experiments for each cell line. Statistical analysis was performed by comparing the cytotoxic effect of doxorubicin alone and in combination with propranolol by unpaired *t* test (two-tailed) (*p ≤ 0.05; **p ≤ 0.01). **(B)** Cell viability assays performed on COSB and DD-1 cells 72 h after incubation with propranolol or propranolol with LysoTracker Deep Red. Data are presented as the means ± S.D. of at least three independent experiments for each cell line. Statistical analysis was performed by comparing the cytotoxic effect of doxorubicin alone and in combination with LysoTracker Deep Red by unpaired *t* test (two-tailed). **(C)** Dot plot representation of the combination index (CI) of propranolol in association with doxorubicin across the cell line panel. Cell viability assays were performed after 72 h of incubation with a range of propranolol and doxorubicin drug concentrations. Untreated cells or cells treated only with propranolol or doxorubicin served as controls and were used to determine the relative LC50 values for each drug in each experiment. The CI values were determined based on the method of Chou and Talalay for all combinations of the drugs tested. Data shown are from a representative experiment from each cell line performed in duplicate. Each set of conditions was tested in at least three independent experiments for each cell line. The gray bar is used to mark the range for drug additivity (0.9 ≤ CI ≤ 1.1).

Because propranolol and doxorubicin both accumulate within lysosomes (20, 26) and are often administered in combination to treat human angiosarcoma (34, 54) and canine hemangiosarcoma (55), we sought to determine if propranolol could potentiate the antiproliferative effects of doxorubicin through reduction of lysosomal doxorubicin entrapment. We first confirmed previous observations that propranolol accumulates in lysosomes by incubating the COSB cell line with LysoTracker Deep Red, a fluorescent dye that is highly selective for acidic organelles. Lipophilic amines, such as propranolol, have been shown to induce the concentration-dependent inhibition of LysoTracker Deep Red accumulation within lysosomes (20). Propranolol decreased the relative fluorescence of LysoTracker Deep Red in COSB cells in a concentration-dependent manner (**Supplemental Figure S3A**). Next, drug combination studies using a viability assay were performed to determine whether propranolol could potentiate the antiproliferative effects of doxorubicin. We treated our collective cell line panel with increasing concentrations of doxorubicin alone or in combination with a fixed concentration of propranolol (50 µM), a concentration well below the determined LC50 values for each line and assessed changes in cell viability 72 h after drug treatment. Propranolol decreased the relative LC50 values to doxorubicin in the COSB, DD-1, ISO-HAS, and SVR cell lines by 8.3-, 3.2-, 1.4-, and 5.7-fold, respectively, when compared to cells treated with doxorubicin alone (**Figure 2A**). The differences between the LC50 values for control and drug treated cells were significant for the COSB (p ≤ 0.05), DD-1 (p ≤ 0.01), and SVR (p ≤ 0.01) cell lines, while the difference for the ISO-HAS line was not significant (p = 0.3115). To confirm that sensitization of the tumor cells to doxorubicin by propranolol occurs through a β-AR-independent mechanism, we treated COSB cells with the S-(−) and R-(+) enantiomers of propranolol, or a reconstituted racemic mixture of the enantiomers. The enantiomers alone or in combination sensitized the cells to doxorubicin to the same extent as cells treated with racemic propranolol or a reconstituted mixture of the enantiomers (**Supplemental Figure S3B**). To further test whether the decrease in the relative LC50 values was due, at least in part, to competition for lysosomal accumulation, we incubated the COSB and DD-1 cell lines with increasing concentrations of doxorubicin with or without LysoTracker Deep Red for 72 h. LysoTracker Deep Red in combination with doxorubicin consistently reduced the relative LC50 values for COSB and DD-1 cells by approximately 1.5-fold when compared to cells treated with doxorubicin alone (**Figure 2B**), although the reductions were not statistically significant. The interaction of propranolol with doxorubicin was then quantified using the method of Chou and Talalay (50). Analyses showed that the combination index (CI) for the association of propranolol with doxorubicin was moderately to highly synergistic in the DD-1, ISO-HAS, and SVR cell lines and moderately synergistic to slightly additive in COSB cells (**Figure 2C**).

**Figure 3:**
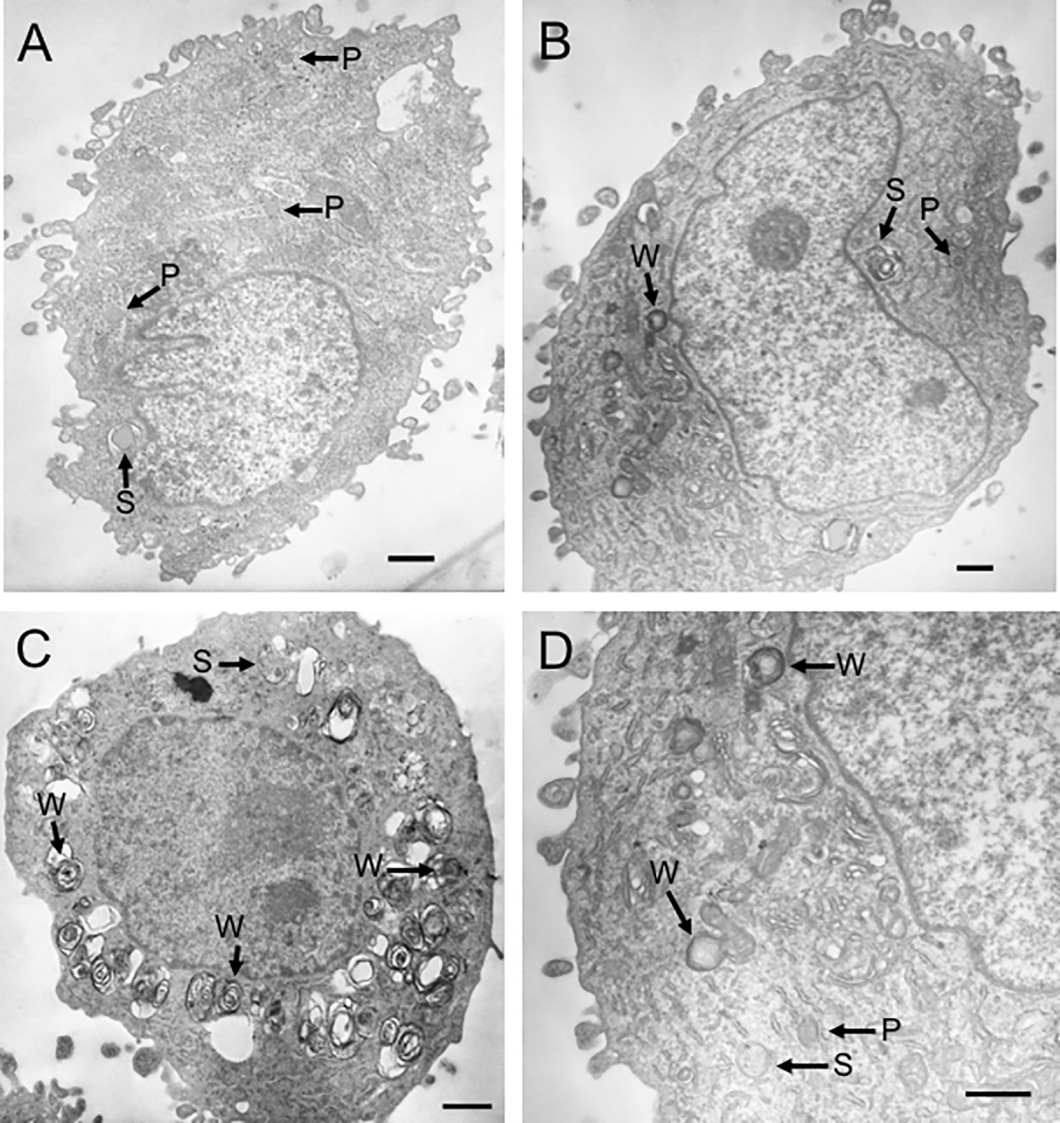
Propranolol increases lysosomal volume. Transmission electron micrographs representative of COSB cells from three different treatment groups. **(A)** Control cell (not exposed to propranolol). The lysosomal system consisted of primary (P) and secondary (S) lysosomes. **(B)** Cell exposed to 50 µM propranolol for 30 days with lipid-laden membrane whorls (W) present. **(C)** Cell exposed to 100 µM propranolol for 30 days. **(D)** Higher magnification view of the cell in panel **(B)** illustrating the three types of lysosomal organelles observed in COSB cells exposed to propranolol. Scale bars = 1 µm. One-way ANOVA was used to determine statistical significance between treatment groups based on the percentage of cytoplasm occupied by the lysosomal organelles. Eleven cells were analyzed per treatment group, and the data are presented in **Table 2** as mean percentage ± S.D.

Because propranolol has been shown to increase both the size and number of lysosomes in fibroblasts after continuous, long-term treatment (26), we sought to confirm that propranolol induced a similar effect in the sarcoma cell lines. We also determined if changes in lysosomal volume and removal of propranolol would alter the sensitivity of cells to doxorubicin. This is relevant to the clinical application of propranolol in combination with doxorubicin because continuous, long-term treatment regimens of propranolol with chemotherapy have been incorporated into treatment protocols for angiosarcoma patients (28–32, 34). We treated COSB cells with 50 µM or 100 µM propranolol every other day for 30 days to mimic long-term drug treatment. Using this approach, cell viability consistently remained above 90% (data not shown). In control cells, the lysosomal system occupied approximately 3.5% of the cell cytoplasm and consisted of primary (P) and secondary (S) lysosomes (**Figure 3A** and **Table 2**). Long-term exposure to 50 µM or 100 µM propranolol significantly (p ≤ 0.001) increased the proportion (both size and number) of lysosomal organelles making up the cell cytoplasm (**Figures 3B-D**). An increase in the number of secondary lysosomes and lysosomes containing lipid-laden membrane whorls (W) was more apparent in cells treated with 100 µM propranolol compared to those treated with 50 µM propranolol, along with a concomitant decrease in primary lysosome number.

**Table 2:** Percentage of cytoplasm occupied by lysosomal organelles.

Control (DMSO)	3.5 +/− 1.1%
50 µM propranolol	6.6 +/− 3.0%
100 µM propranolol	16.0 +/− 4.8%

**Figure 4:**
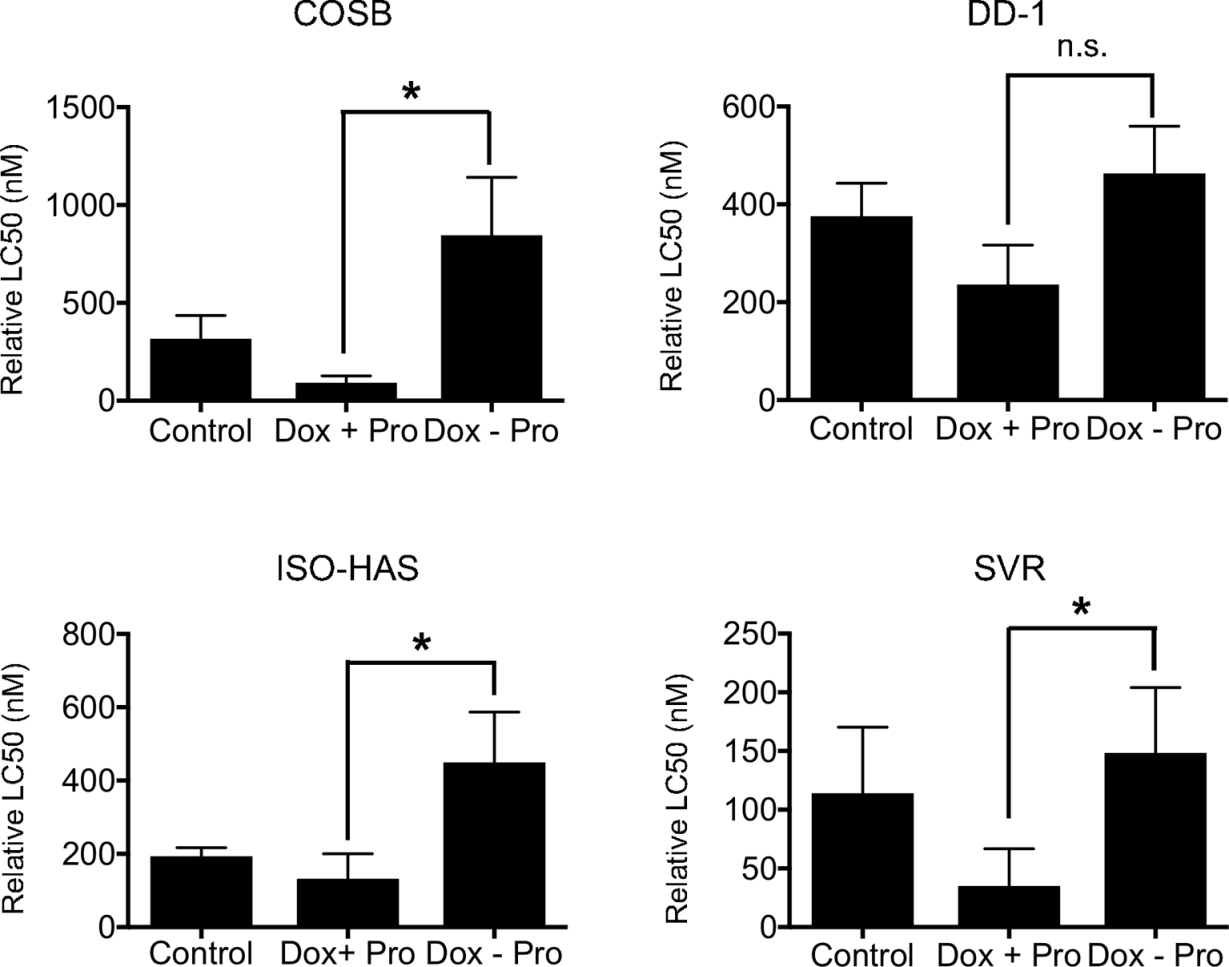
Effect of long-term propranolol treatment on doxorubicin cytotoxicity. COSB, DD-1, ISO-HAS, and SVR cells were treated with 50 µM propranolol every other day for two weeks, and cell viability assays were carried out 72 h after incubation with doxorubicin, doxorubicin with propranolol, or doxorubicin alone after propranolol was removed. Percent cell viability was determined by comparison to untreated control cells. The LC50 values were determined using Prism software. Data are presented as means ± S.D. of at least three independent experiments. Statistical analysis was performed by comparing the cytotoxic effect of doxorubicin (Control), doxorubicin with propranolol (Dox + Pro), or doxorubicin after the removal of propranolol (Dox – Pro) using one-way ANOVA.*p ≤ 0.05, n.s., no significance.

To determine whether expansion of the lysosomal system alters the sensitivity of cells to doxorubicin, we treated the cell line panel with 50 µM propranolol every other day for two weeks to expand the lysosomal volume. After two weeks, cells were treated with propranolol in the presence of increasing concentrations of doxorubicin, and the cell viability was determined after 72 h of treatment. We also removed propranolol from the cells during the 72-hour incubation period with doxorubicin to address the hypothesis that the expanded lysosomal volume of the propranolol-treated cells would increase the capacity of cells to sequester doxorubicin when propranolol was removed. Removal of propranolol should then lead to an increase in the overall resistance of the cells to the chemotherapy. Long-term exposure to propranolol sensitized all of the cell lines to doxorubicin when compared to cells treated with doxorubicin alone (**Figure 4**). In contrast, removal of propranolol from the assay during doxorubicin treatment led to a significant (p ≤ 0.05) increase in the LC50 values for 3 of the 4 cell lines when compared to the LC50 values from cells treated with doxorubicin and propranolol in combination. Collectively, our results indicate that lysosomal volume is associated with the sensitivity of the cell lines to doxorubicin. Our results also suggest that propranolol competes effectively with doxorubicin for the lysosomal space, during both short- and long-term exposures to propranolol, rendering the tumor cells more sensitive to the chemotherapy.

### Propranolol Increases the Intracellular Levels of Doxorubicin and Enhances the DNA Damage Response

**Figure 5:**
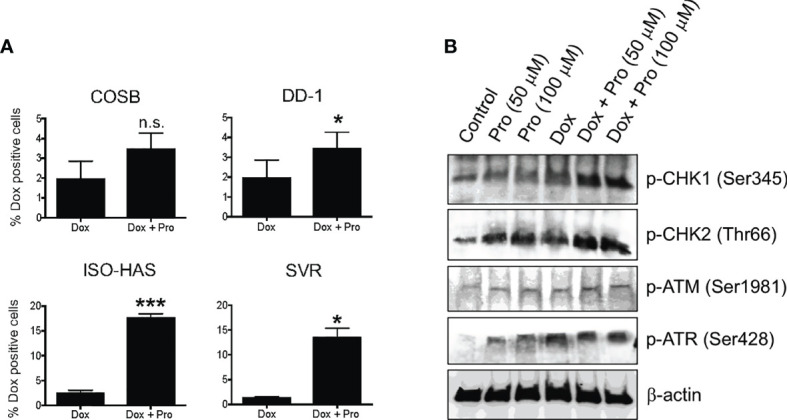
Propranolol alters the cellular retention of doxorubicin. **(A)** Cytotoxic effects of doxorubicin in the presence of propranolol. The percentage of doxorubicin positive cells was assessed by flow cytometry. Data are presented as the means ± S.D of at least two independent experiments with each treatment performed in duplicate. Statistical significance was determined by unpaired *t* test (two-tailed) (*P < 0.05; ***P < 0.001), n.s., no significance. **(B)** Immunoblot showing changes in DNA damage response proteins in COSB cells in response to different treatments with propranolol and doxorubicin.

Propranolol has been shown to increase the intracellular accumulation of vinblastine in neuroblastoma cells (39), prompting us to investigate whether propranolol also promotes the accumulation of doxorubicin. We evaluated the impact of propranolol on doxorubicin retention across our cell line panel by treating cells with doxorubicin for 1 h followed by treatment with propranolol for 18 h. We chose an 18 h exposure period to provide sufficient time for doxorubicin efflux, allowing easier visualization of changes in the drug’s retention under different conditions (15). Propranolol significantly (p ≤ 0.05) increased the number of doxorubicin positive cells in the DD-1, ISO-HAS, and SVR cell lines (**Figure 5A**). Overall, the percentage of positive cells increased by approximately 2- to 10-fold (**Supplemental Figure S4A**). To determine whether the increased doxorubicin retention was through a β-AR independent mechanism, we treated the COSB cell line with the S-(−) and R-(+) enantiomers of propranolol or a reconstituted mixture of the enantiomers. Similar increases in intracellular doxorubicin retention were observed under all treatment conditions (**Supplemental Figure S4B**). To further rule out a contribution from β-ARs, we treated the ISO-HAS and SVR cell lines with levels of propranolol within a concentration range appropriate to the affinities of the drug for β1- and β2-ARs, 100 nM and 1 µM, respectively (53). The two cell lines were chosen because of the significant increase observed in the number of doxorubicin positive cells in the presence of propranolol (**Figure 5A**). Increased retention of doxorubicin was not observed in either cell line using the lower concentrations of propranolol (**Supplemental Figure S4C**).

Because propranolol increased the intracellular levels of doxorubicin and doxorubicin induces DNA damage, we surmised that propranolol might enhance DNA damage response pathways. Treatment of COSB cells with propranolol or doxorubicin enhanced DNA damage pathway activation based on the increased levels of phosphorylation observed in the DNA damage response proteins ATR, CHK1, and CHK2 (**Figure 5B**). Additional increases in phosphorylation were observed for CHK1 and CHK2 when cells were pretreated with propranolol followed by treatment with doxorubicin. A slight increase in phospho-ATM was observed under all treatment conditions when compared to untreated controls.

### Propranolol Inhibits Drug Efflux

Based on the increased retention of doxorubicin in the presence of propranolol, we undertook a functional analysis of ATP-binding cassette (ABC) transporter activity to determine if propranolol inhibited drug efflux by blocking transporter function. To evaluate efflux, we used DCV, a viable dye eliminated from cells by several ABC efflux transporters, including those encoded by the ABCB1 (P-glycoprotein or P-gp) and ABCG2 (encoding the breast cancer resistance protein or BCRP) genes (56, 57). Doxorubicin is a substrate for both the P-gp and BCRP transporters (58, 59). We previously verified the expression of these two transporters in the COSB and DD-1 cell lines along with the ability of these cells to efflux DCV (15). Furthermore, previous studies have shown that propranolol is a potent inhibitor of P-gp (60).

**Figure 6:**
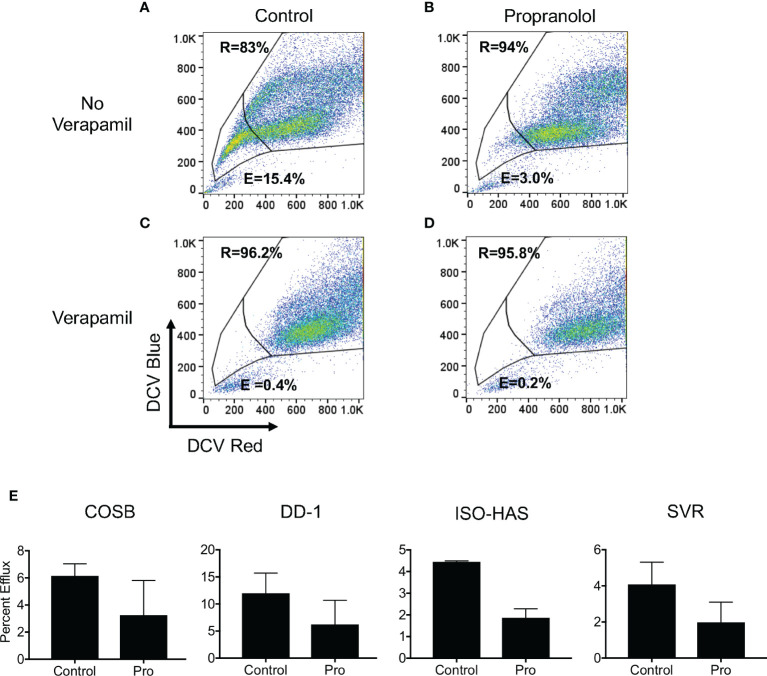
Propranolol inhibits the cellular efflux of DyeCycle Violet. **(A)** Representative experiment showing the identification of cell populations capable of effluxing (indicated by “E” in each panel) and retaining (indicated by “R” in each panel) DyeCycle Violet in DD-1 cells and **(B)** inhibition of dye efflux by verapamil. Analysis of the effects of **(C)** propranolol and **(D)** propranolol and verapamil on DCV efflux and retention. Propidium iodide was added immediately before examination of the samples by flow cytometry in order to exclude dead cells from the analysis. **(E)** Summary of the changes in dye efflux in response to propranolol in all four cell lines. The complete dye efflux and dye retention data for the four cell lines are summarized in **Supplementary Table 3**. The results for each line are presented as the means ± S.E. of at least three independent experiments for each line.

We first defined dye efflux (E) and dye retention (R) within subpopulations of each cell line by observing efflux in the presence and absence of verapamil, a broad inhibitor of ABC transporter activity (57, 59). Efflux and retention populations for the DD-1 cell line from a representative experiment are shown as an example (**Figures 6A, B**). We then used this gating strategy for all cell lines to determine the effects of propranolol on DCV efflux. Propranolol consistently reduced dye efflux across the cell lines by approximately 1.8- to 2.4-fold (**Figures 6C–E**). The dye effluxing and retaining cell population data for each line are fully summarized in **Supplemental Table 3**. We also examined the effects of the S-(−) and R-(+) enantiomers of propranolol on DCV efflux in DD-1 cells. Substantial differences in dye efflux were not observed between racemic propranolol, equimolar concentrations of each enantiomer, or a reconstituted racemic mixture (**Supplemental Figures S5A, B**). Taken together, these data suggest that propranolol increases the intracellular levels of doxorubicin by blocking ABC transporter drug efflux pumps through a β-AR-independent mechanism.

## Discussion

Drug repurposing or repositioning is a strategy for identifying new uses for already approved drugs or compounds that fall outside the scope of the originally intended indication. Our investigation identified two potential mechanisms by which propranolol potentiates the anti-proliferative properties of doxorubicin *in vitro*. First, our data suggest that propranolol sensitizes cells to doxorubicin by reducing its accumulation in lysosomes, thereby increasing its concentration at its site of action in the cell nucleus. By taking advantage of the β-AR receptor-active S-(−) and -inactive R-(+) enantiomers of propranolol, we discovered that the potentiating effect of propranolol is not attributable to its stereoselective interactions with β-ARs. Rather, its action appears to stem from the physicochemical properties of propranolol as a lipophilic organic base. Viability assays with propranolol and LysoTracker Deep Red reduced the relative LC50 values of doxorubicin on multiple cell lines. Expansion of the lysosomal volume by propranolol and its abrupt removal led to increases in tumor cell resistance to doxorubicin. These observations are consistent with previous data showing that propranolol synergizes with multiple lysosomotropic chemotherapeutics (25, 39, 61), and their accumulation in lysosomes (20, 26). Collectively, our results indicate that lysosomal volume is associated with the sensitivity of angiosarcoma and hemangiosarcoma cells to doxorubicin and suggest that propranolol synergizes with doxorubicin by competing with the chemotherapeutic for lysosomal accumulation.

Second, we demonstrated that propranolol increases the intracellular accumulation of doxorubicin, as we observed an increase in the number of doxorubicin-positive cells after treatment with propranolol. This result is in line with previous observations showing that propranolol increased the intracellular accumulation of the lysosomotropic plant alkaloid, vincristine, in neuroblastoma cell lines (39) and doxorubicin in sarcoma cell lines (62). Using a DCV efflux assay, we found that propranolol reduced dye efflux across all of the cell lines used in this study. Because DCV and doxorubicin can be effluxed from these cells by P-gp (57, 59), and propranolol is a potent inhibitor of this transporter (60, 63, 64), our observations support the hypothesis that propranolol blocks the efflux of doxorubicin from cells through inhibition of P-gp and possibly other transporters. Taken together, our data show that propranolol increases the intracellular accumulation of doxorubicin and support previous studies showing that propranolol blocks drug efflux. This mechanism also supports our observations that propranolol in combination with doxorubicin enhances the DNA damage response, as an enhanced response may be due to increased accumulation of doxorubicin in the cell nucleus.

The competition between two different lysosomotropic drugs and their respective lysosomal accumulation depends on two physicochemical properties, the basic pKa (acid dissociation constant for the conjugated acid of the weak base) and logP (the logarithm of the partition coefficient of a compound between octanol and water, representing membrane permeability) (20, 65). These properties influence drug accumulation by affecting the extent of lysosomal trapping and passive membrane permeation, respectively. Lysosomotropic amine-containing drugs are generally thought to inhibit the lysosomal uptake of each other based on the ability of these drugs to increase the lysosomal pH (66–71); however, other studies suggest that some drugs may induce a transient increase in pH followed by restoration of lysosomal pH after prolonged compound sequestration (26, 65, 72). Previous reports have shown that propranolol synergizes with lysosomotropic chemotherapeutics *in vitro*, including vinblastine, paclitaxel, doxorubicin, and the lysosomotropic tyrosine kinase inhibitor, sunitinib (32, 38, 39, 61). All of these compounds carry a basic moiety with basic pKa values ranging from 8.7 to 10.9, and all are lipophilic with calculated logP values spanning from 0.92 to 3.13 (25, 73).

Our results showed moderate to high synergy between propranolol and doxorubicin in the DD-1, ISO-HAS, and SVR cell lines and moderate synergy in COSB cells. In contrast to our findings, only marginal effects with doxorubicin were reported in synergy studies using transformed endothelial cells as a model of angiosarcoma (32). Instead, propranolol strongly potentiated the anti-proliferative effects of vinblastine in these cells. These results led to the design of a pilot study combining oral propranolol and metronomic vinblastine in a small number of patients with advanced metastatic or recurrent angiosarcoma (32). In contrast to the synergistic responses observed with lysosomotropic agents, the non-lysosomotropic, pyrimidine analogue, 5-fluorouracil, produced largely antagonistic effects in the majority of the cell lines analyzed (38). An interpretation of these data is that new combinatorial approaches for the treatment of angiosarcoma and hemangiosarcoma should focus on the addition of propranolol to hydrophobic, weak base chemotherapeutics. Screening additional chemotherapy drugs in an expanded panel of angiosarcoma and hemangiosarcoma cell lines and translation of these findings to xenograft models of angiosarcoma (33) and hemangiosarcoma (40) may further refine the identification of effective combinatorial strategies for clinical translation.

In addition to altering intracellular drug concentrations and distribution, propranolol may also impact other mechanisms used by cancer cells to induce resistance, such as altered cell cycle regulation and increased DNA repair. Evidence suggests these processes may be regulated, at least in part, by the microphthalmia/transcription factor E (MiT/TFE) family of transcription factors (MITF, TFE3, TFEB, and TFEC), which control transcriptional programs for autophagy and lysosome biogenesis to sustain cancer cell growth and survival under stress conditions (74). Hydrophobic weak base compounds, including doxorubicin and propranolol, trigger lysosomal stress and lysosomal biogenesis through the activation of the transcription factor TFEB by promoting its migration from the cytoplasm to the nucleus (25, 26). This TFEB-mediated, drug induced activation of lysosomal biogenesis results in a significant increase in lysosome number as well as size, which we confirmed through long-term exposure of COSB cells to propranolol.

TFEB has also been shown to activate p53, a key transcriptional regulator of the DNA damage response that activates essential genes involved in cell cycle arrest, DNA repair, and ultimately apoptosis (75–77). We previously showed that propranolol increased the expression of p53 in angiosarcoma and breast cancer cells *in vitro* and in the tumor tissue from a breast cancer patient treated with propranolol (33, 78). Based on the results from our synergy studies and our observation that propranolol increased the number of doxorubicin-positive cells in our assays, we tested the idea that propranolol would enhance DNA damage response pathways. Compared to each drug alone, our results showed enhanced DNA damage pathway activation by propranolol and doxorubicin based on the increased levels of phosphorylation observed in DNA damage response proteins. At lower drug concentrations in combination or individually, TFEB may contribute to cell survival by blocking the cell cycle and facilitating DNA repair. Under conditions of heightened stress or prolonged DNA damage when cells are treated with the drugs in combination, cells may further activate p53, overcoming the apoptotic threshold and switching the p53 cell fate from arrest to apoptosis (77). Future studies should evaluate changes in the expression of p53 in response to different propranolol combinations with different genotoxic drugs and lysosomotropic chemotherapeutics and also consider the p53 status of each cell line.

The rare occurrence and heterogeneity of angiosarcoma present a logistical challenge for performing randomized trials to evaluate new treatment approaches for these patients. In contrast, canine hemangiosarcoma occurs frequently in dogs at a rate sufficient to power clinical trials, yet it closely models the genetic landscape and the histopathology of human angiosarcoma and follows a similar clinical course (11, 79). Our findings showing that propranolol sensitizes canine hemangiosarcoma and human angiosarcoma cell lines to doxorubicin have important implications for improving treatment outcomes in both species as identical chemotherapeutics and adrenergic antagonist are used in veterinary and human medicine, allowing for drug repurposing in the canine disease setting. Furthermore, the compressed course of cancer progression in dogs (months versus years), the ability to carry out treatment in the setting of an intact immune system, and the heterogeneity of canine hemangiosarcoma allows for the timely assessment of potential new therapies to treat angiosarcoma (80).

In summary, our study suggests that propranolol synergizes with doxorubicin by decreasing the lysosomal sequestration and the cellular efflux of doxorubicin while increasing its intracellular concentration. These changes may allow for the higher accumulation of doxorubicin in the cell nucleus, leading to prolonged cell stress and apoptosis. Because lysosomal sequestration is a common phenomenon of weakly basic amines, propranolol could be repurposed with chemotherapeutic regimens for the treatment of other cancers, including sarcomas. Finally, we propose that the repurposing and clinical translation of the R-(+) enantiomer would spare patients from many of the common side effects associated with racemic propranolol acting at β-ARs in the heart, airways and other locations (e.g. negative inotropic and chronotropic effects, bronchospasm, changes in glucose and lipid metabolism), while maintaining its therapeutic benefit in cancer chemotherapy. The only side effect reported for R-(+) propranolol in humans is a small reduction in thyroxine to triiodothyronine conversion, which appears to be more pronounced in hypothyroid patients (81). Recent discoveries showing the R-(+) enantiomer is also effective for treating benign vascular diseases (82, 83) suggest that it might be more broadly repurposed for the treatment of other diseases.

## Data Availability Statement

The original contributions presented in the study are included in the article/**Supplementary Material**. Further inquiries can be directed to the corresponding author.

## Author Contributions

JS, JK, CA, CW, and DK performed the experiments. JS, JK, DB, JT, BB, and ED designed the experiments and wrote the paper. AK, JT, BB, and ED supervised the project, and BB and ED conceived of and obtained funding for the project. All authors contributed to the article and approved the submitted version.

## Funding

This work was supported by grants from Morris Animal Foundation (#D17CA-059 and #D18CA-017 to ED) and the Sarcoma Foundation of America (Dr. Richard and Valerie Aronsohn Memorial Research Award to ED and BB). The National Institutes of Health (NIH) Comprehensive Cancer Center Support Grant to the Masonic Cancer Center, University of Minnesota (P30 CA077598) provided support for the flow cytometry core and services. The authors would like to acknowledge donations to the Animal Cancer Care and Research Program of the University of Minnesota.

## Conflict of Interest

The authors declare that the research was conducted in the absence of any commercial or financial relationships that could be construed as a potential conflict of interest.
