# Supplementary material for: Propranolol Sensitizes Vascular Sarcoma Cells to Doxorubicin by Altering Lysosomal Drug Sequestration and Drug Efflux
Source: Front Oncol. 2021 Feb 1;10:614288. doi: 10.3389/fonc.2020.614288 (PMC7882688; doi:10.3389/fonc.2020.614288)
Supplement: Supplementary file 1 [file DataSheet_1.pdf]

Figure S1

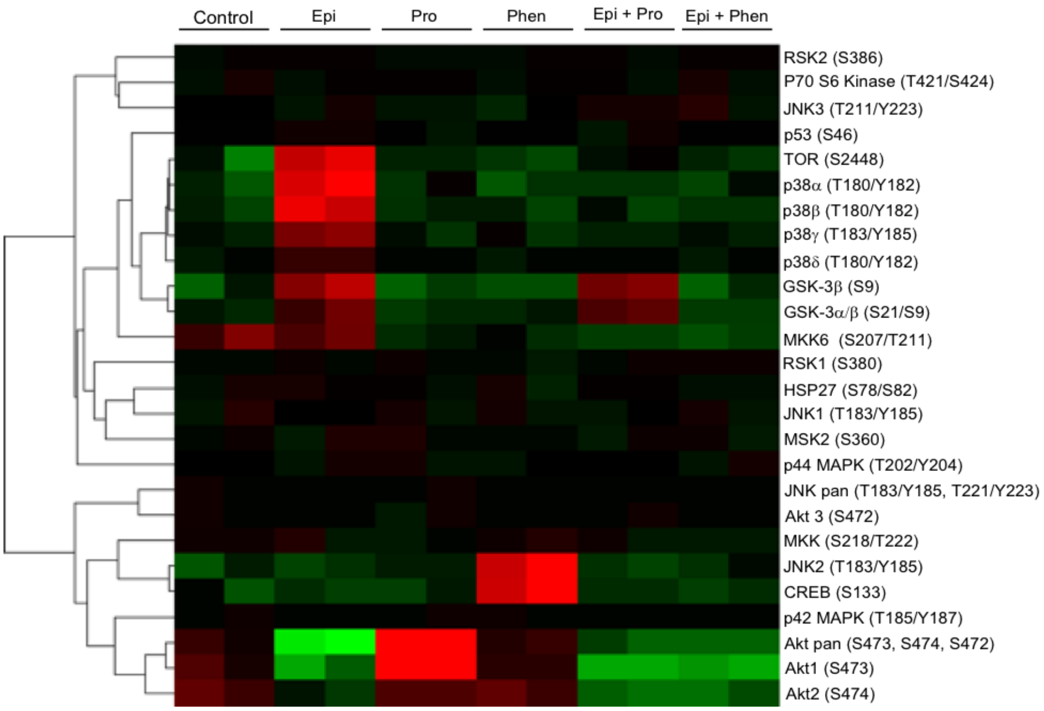

Supplemental Figure S1. Evaluation of adrenergic receptor signaling pathways in COSB cells in response to agonist and antagonist stimulation. Heatmap depicting phosphor-antibody array results showing the changes in the phosphorylation status of 26 kinases in COSB cells treated for 30 minutes with epinephrine (Epi; 1 μM), propranolol (Pro; 100 nM), or phentolamine (Phen; 100 nM) (red=increased phosphorylation, green=decreased phosphorylation).

Figure S2

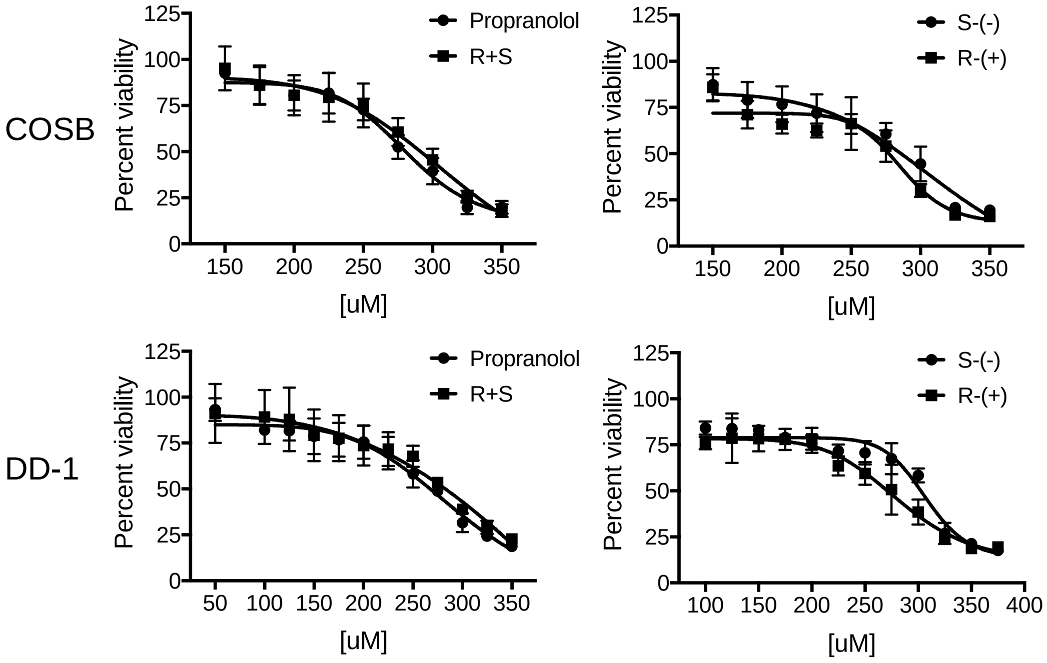

Supplemental Figure S2. Propranolol and its enantiomers reduce hemangiosarcoma cell viability. Cell viability profiles of hemangiosarcoma cells treated with propranolol or the drug enantiomers. The panels show curve fitting of concentration-dependent inhibition of COSB (top) and DD-1 (bottom) cell viability with increasing concentrations of propranolol, the S-(-) and R-(+) enantiomers, or a reconstituted racemic mixture of the enantiomers. The percent cell viability was determined 72 hours after drug treatment using an MTS assay, and the percent viability was calculated by comparing the viability to untreated controls. The LC50 values for propranolol and the enantiomers in the cell lines are presented in Table 1. Each concentration was tested in triplicate or quadruplicate for each assay, and the curves are representative data from assays performed at least three times for each cell line.

Figure S3

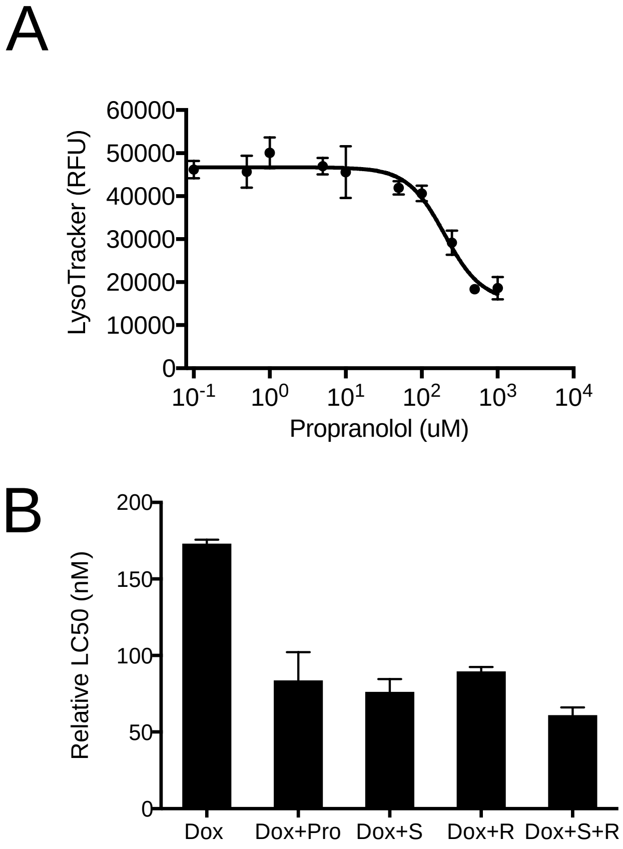

Supplemental Figure S3. Propranolol and its enantiomers synergize with doxorubicin to reduce cell viability. (A) The effect of propranolol on LysoTracker Deep Red fluorescence in COSB cells. Cells were incubated with 50 nM LysoTracker Deep Red and increasing concentrations of propranolol, and the relative fluorescence (ex/em 647/668) was determined after 30 minutes. Data is representative of three experiments. **(B)** COSB cells were incubated with 50 μM propranolol, the S-(-) and R-(+) enantiomers, or a racemic mixture in combination with increasing concentrations of propranolol. The percent cell viability was determined using an MTS assay and was calculated after 72 hours of drug treatment by comparing the viability to untreated controls. The LC50 values were determined using Prism software.

Figure S4

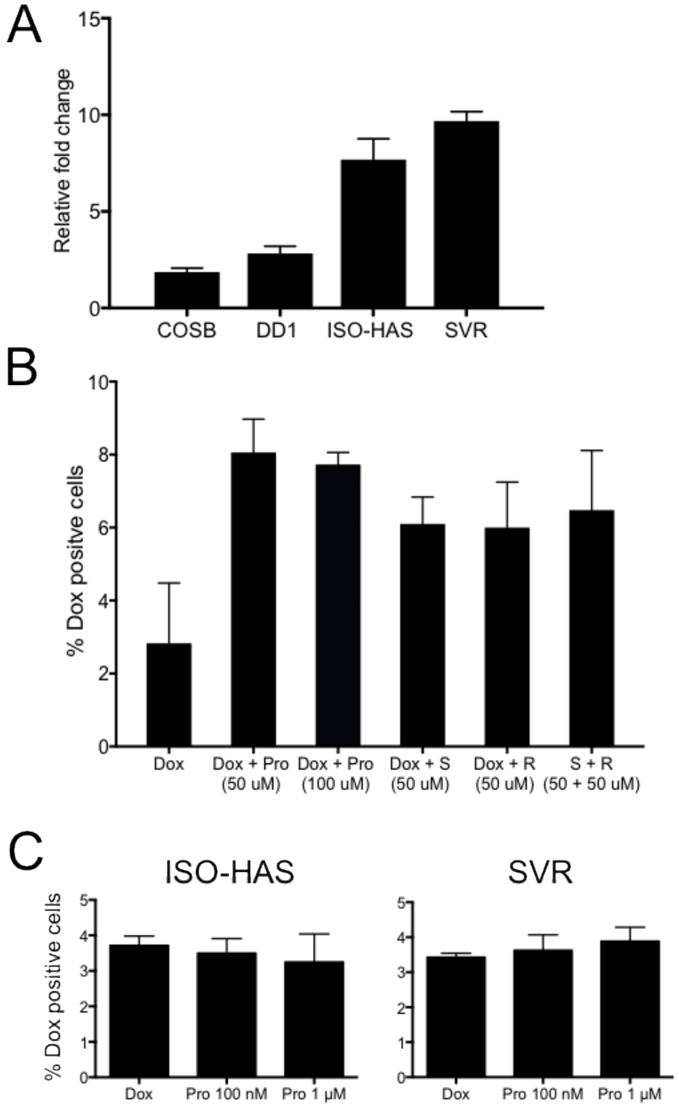

Supplemental Figure S4. Propranolol promotes doxorubicin retention via a β-AR-independent mechanism. (A) Percent increase in the number of doxorubicin positive cells from Figure 5A represented as fold change. **(B)** Assessment of the percent doxorubicin positive cells in the COSB cell line after treatment with propranolol, the S-(-) and R-(+) enantiomers, or a racemic mixture of the enantiomers. **(C)** Assessment of the percent doxorubicin positive cells in the ISO-HAS and SVR cell lines after treatment with doxorubicin concentrations of propranolol using concentrations (100 nM and 1 μM) appropriate to the affinities of propranolol for β1- and β2-ARs.

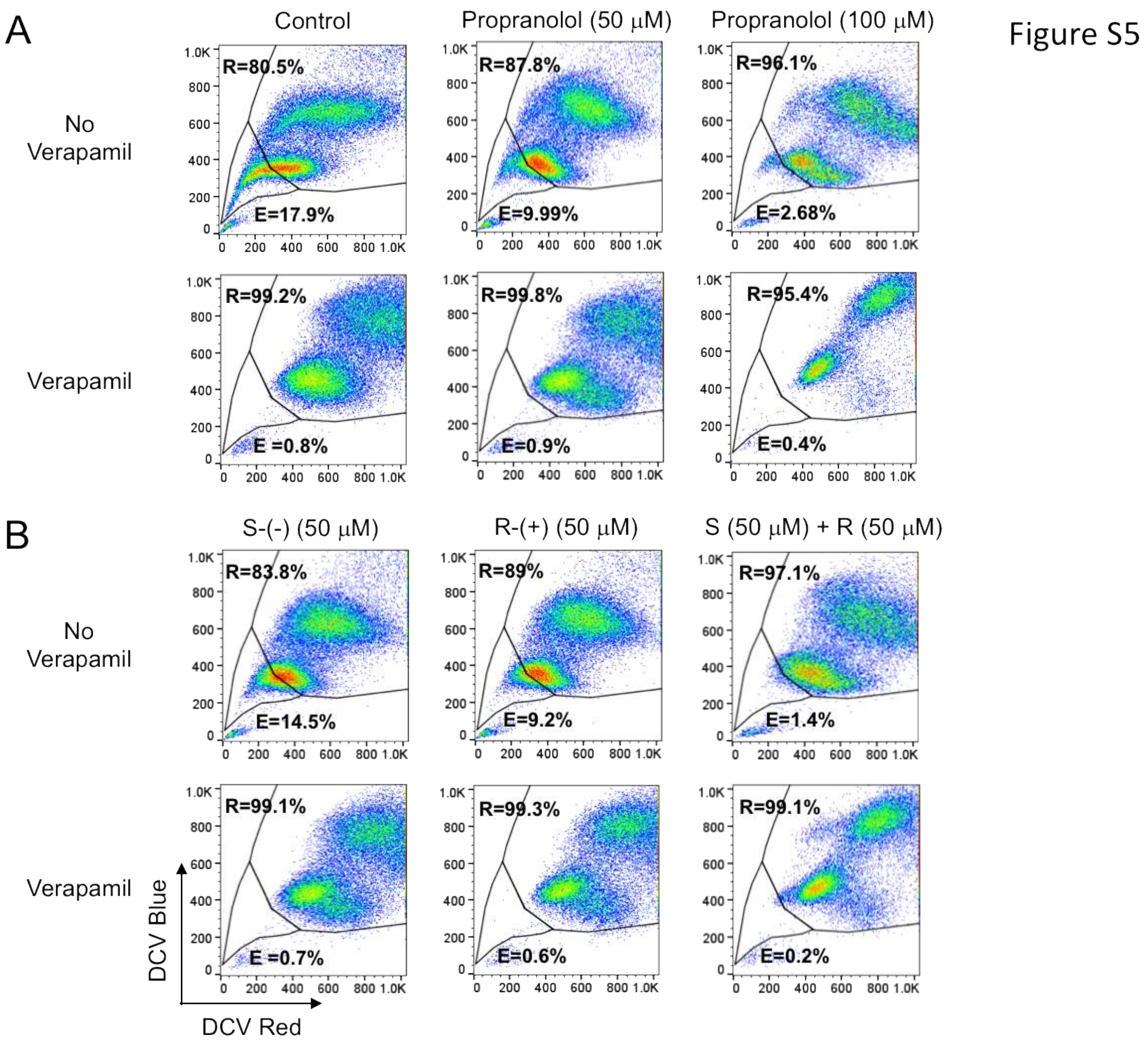

Supplemental Figure S5. Propranolol inhibits the efflux of DyeCycle Violet via a receptor-independent mechanism. Analysis of the effects of **(A)** propranolol and **(B)** the enantiomers of propranolol on DCV efflux and retention. Controls for verapamil are shown to establish gating for the effluxing populations. Propidium iodide was added immediately before examination of the samples by flow cytometry in order to exclude dead cells from the analysis. The results are representative of three independent experiments.
